# Fatal hypoglycemia with ciprofloxacin in a dialysis patient: A case report

**DOI:** 10.1002/ccr3.3871

**Published:** 2021-03-12

**Authors:** Aisa Matoi, Mana Taguchi, Shinichi Nishi

**Affiliations:** ^1^ Intensive Care Unit Hyogo College of Medicine Nishinomiya Japan

**Keywords:** adverse event, ciprofloxacin, fatal hypoglycemia, fluoroquinolones, renal dysfunction

## Abstract

In patients with renal dysfunction, it is important to avoid prescribing fluoroquinolones including ciprofloxacin.

## Graphical abstract

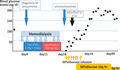

## 1 INTRODUCTION

Fluoroquinolones are widely used for various infections because of their broad antibacterial spectrum and usefulness, particularly in Japan. Fluoroquinolones have been reported to have complications such as dysglycemia,^1^ cardiovascular toxicity,^2^ and tendinopathy,^3^ but we poorly recognize that. In fact, the use of fluoroquinolones should be limited from the complications and the antimicrobial resistance perspective. There are many reports of hypoglycemia caused by fluoroquinolones, especially gatifloxacin and levofloxacin, but relatively few reports of hypoglycemia caused by ciprofloxacin. Here, we report a case of fatal hypoglycemia associated with ciprofloxacin administration in a dialysis patient.

## 2 CASE PRESENTATION

**FIGURE 1:**
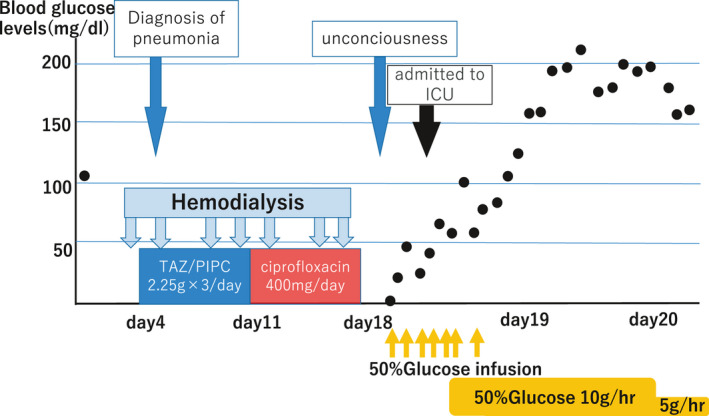
Administration of antibacterial drugs and blood sugar levels after admission. TAZ/PIPC= tazobactam‐piperacillin

A 73‐year‐old man with end‐stage renal disease on hemodialysis and atrial fibrillation was admitted to hospital for surgery for spondylosis. He had end‐stage renal disease due to diabetic nephropathy, but he was not taking diabetic drugs for decades. Four days after admission, he developed fever, and pneumonia was suspected by computed tomography (CT) examination. Intravenous tazobactam‐piperacillin was administered. After 7 days of thrice‐daily administration of 2.25 g tazobactam‐piperacillin, the antibiotic was changed to ciprofloxacin for suspected atypical pneumonia. After intravenous administration of 400 mg ciprofloxacin once daily for 7 days, he was found to be in a state of unconsciousness on the morning of a dialysis day. Blood test revealed blood glucose level of 1 mg/dL. He was administered glucose by infusion, but hypoglycemia and altered mental status persisted and he was admitted to the intensive care unit. There, he received glucose infusions and his blood glucose level normalized, although his consciousness did not improve (see Figure 1). The head nonenhanced CT examination revealed no intracranial lesion, and the patient was diagnosed with hypoglycemic encephalopathy.

An endocrine test (Table 1) revealed that counter‐regulatory hormones were elevated, but C‐peptide and insulin levels were high and normal, respectively (C‐peptide 3.68 ng/mL, insulin 8.01 μIU/mL), suggesting hyperinsulinemic hypoglycemia. Chest‐abdominal CT examination did not reveal insulinoma.

**TABLE 1:** Endocrine test during an episode of hypoglycemia

C‐peptide (ng/mL)	3.68 (0.61**‐**2.09)	GH^¶^ (ng/mL)	6.98 (<2.47)
Insulin (μIU/mL)	8.01 (1.84**‐**12.2)	ACTH^††^ (pg/mL)	28.7 (7.2**‐**63.3)
Insulin antibody	Negative	Cortisol (μg/dL)	13.6 (6.24**‐**18)
free T_3_^†^ (pg/mL)	2.68 (2.3**‐**4.0)	Adrenaline (pg/mL)	237 (<100)
free T_4_^‡^ (ng/dL)	1.20 (0.9**‐**1.7)	Noradrenaline (pg/mL)	651 (100**‐**450)
TSH^§^ (μIU/mL)	16.4 (0.5**‐**5.0)	Dopamine (pg/mL)	86 (<20)

Note

^†^free T3=free thyroxine 3; ^‡^free T4=free thyroxine 4; ^§^TSH=thyroid stimulating hormone; ^¶^GH=growth hormone; ^††^ACTH=adrenocorticotropic hormone. Numbers in parentheses are reference values.

Subsequently, his consciousness did not improve, and he died 73 days after admission due to aspiration pneumonia.

## 3 DISCUSSION

Fluoroquinolones are antibiotics widely used in the treatment of common bacterial infections in patients due to a broad spectrum of antibacterial activity and high oral bioavailability. But various adverse effects have been reported, such as dysglycemia,^1^ cardiovascular toxicity,^2^ and tendinopathy.^3^ In July 2016, the US Food and Drug Administration approved safety labeling changes for fluoroquinolones to enhance warnings about their association with disabling and potentially permanent side effects and to limit their use in patients with less serious bacterial infections.^4^ Nevertheless, we do not recognize fully the harmful effects of fluoroquinolones.

Although there are many reports of hypoglycemia caused by fluoroquinolones, ciprofloxacin is considered to have a lower risk of hypoglycemia compared with other fluoroquinolones, as indicated in previous observational studies.^1^, ^5^ There are no previous case reports on severe hypoglycemia because of ciprofloxacin that resulted in serious sequelae such as disorders of consciousness and death, as in the present case.

The mechanism of insulin secretion is as follows: ATP‐sensitive K^+^ channels on pancreatic β‐cells are suppressed by increased blood sugar, while depolarization is caused by an increase in intracellular K^+^ concentration, which causes the opening of voltage‐dependent Ca^2+^ channels and Ca^2+^ influx into the cell body, causing insulin secretion. Fluoroquinolones are believed to promote insulin secretion by suppressing ATP‐sensitive K^+^ channels on pancreatic β‐cells, thereby causing hypoglycemia.^6^ This is consistent with observations in the present case, whereby blood insulin and C‐peptide levels were not suppressed despite hypoglycemia.

Ciprofloxacin is less likely to cause hypoglycemia than other fluoroquinolones. In contrast, a study in noninsulin‐dependent diabetic patients showed ciprofloxacin decreased blood glucose level and increased blood insulin level compared with placebo, although it did not lead to hypoglycemia.^7^ Additionally, it was reported that inhibition of CYP3A4 by ciprofloxacin increases blood levels of sulfonylureas, and its drug interactions may cause hypoglycemia.^8^ A recent study also suggests that ciprofloxacin can cause hypoglycemia even in nondiabetic and previously healthy patients.^9^

The present case was a dialysis patient with end‐stage renal disease due to diabetic nephropathy, who was not taking drugs for diabetes in combination with other medications. Hypoglycemia develops easily in chronic kidney disease patients because of various metabolic abnormalities,^10^ such as reduction of insulin excretion and destruction in the kidney, reduction of gluconeogenesis in kidney and liver, and decrease of counter‐regulatory hormone secretion. Moreover, because fluoroquinolones are primarily excreted by the kidney, administration of fluoroquinolones may cause increased drug accumulation and concentration in patients with renal dysfunction. Regarding the present case, the marked hypoglycemia was potentially caused by promotion of insulin secretion by ciprofloxacin in addition to the state of chronic renal failure.

To our knowledge, there are five reported cases of severe sequelae such as sustained consciousness disorder and death because of hypoglycemia caused by fluoroquinolones.^11^, ^12^, ^13^, ^14^, ^15^ All were because of levofloxacin, not ciprofloxacin. Three of five patients were taking concomitant medications that can cause hypoglycemia, such as insulin and sulfonylureas, and four of five patients had renal dysfunction. It is possible that renal dysfunction with concomitant use of diabetes drugs may contribute to aggravation of hypoglycemia.

Previous studies showed that risk factors of fluoroquinolone‐related hypoglycemia include old age, renal dysfunction, diabetes, and concomitant use of hypoglycemic drugs.^1^, ^16^ The incidence of hypoglycemia in the absence of diabetes drugs is rare, and there is no evaluation of risk factors for onset and severity of hypoglycemia because of fluoroquinolones in nondiabetic patients. However, the identification of hypoglycemia in nondiabetic patients tends to be delayed, which may lead to unfortunate consequences. It is necessary to evaluate the risk factors for hypoglycemia caused by fluoroquinolones in nondiabetic patients. Renal dysfunction may be considered a risk factor because of the mechanism.

## 4 CONCLUSION

Although hypoglycemia caused by ciprofloxacin is rare, it can cause fatal complications, as in the present case. Therefore, it should be administered carefully. Hypoglycemia can potentially develop because of renal dysfunction which is suggested to be a risk factor for aggravation. It is important to avoid prescribing fluoroquinolones, including ciprofloxacin, for patiens with renal dysfunction given the risk of hypoglycemia.

## CONFLICT OF INTEREST

The authors declare no conflicts of interest associated with this manuscript.

## AUTHOR CONTRIBUTIONS

AM: involved in conceptualization, visualization, and writing—original draft. MT: involved in writing—review and editing. SN: supervised the study. All authors: read and approved the final manuscript.

## ETHICAL APPROVAL

We have obtained an informed consent from the patient's family.

## Data Availability

No data were analyzed, reused, and generated in this case report.
